# Case Report: Successful Management of a Compressive Intraspinal *Coccidioides* Species Granuloma in a Cat

**DOI:** 10.3389/fvets.2021.801885

**Published:** 2022-01-03

**Authors:** Hannah Dowdy, Jason E. Evans, Jared A. Jaffey, Kathryn L. Wycislo, Jason D. Struthers, Eric T. Hostnik

**Affiliations:** ^1^Department of Specialty Medicine, College of Veterinary Medicine, Midwestern University, Glendale, AZ, United States; ^2^Department of Pathology, College of Veterinary Medicine, Midwestern University, Glendale, AZ, United States; ^3^Department of Veterinary Clinical Sciences, Veterinary Medical Center, Ohio State University, Columbus, OH, United States

**Keywords:** coccidioidomycosis, feline, neurosurgery, fluconazole, disseminated, central nervous system

## Abstract

A 9-year-old, neutered male, domestic shorthair cat from Arizona, was presented for evaluation of a 7-day history of hind limb paraparesis that progressed to paraplegia. There was no history of respiratory abnormalities. Neurologic examination supported localization of a T3-L3 myelopathy. Computed tomography (CT) revealed an expansile widening of the spinal canal dorsal to L4 associated with a strongly contrast-enhancing mass. Moreover, CT series of the thorax revealed a diffuse miliary pulmonary pattern, as well as tracheobronchial, sternal, and cranial mediastinal lymphadenomegaly. Transthoracic lung lobe and sternal lymph node fine needle aspiration revealed pyogranulomatous inflammation with *Coccidioides* spp. spherules and endospores. A suspected diagnosis of spinal coccidioidomycosis was made; fluconazole (10.9 mg/kg PO q12h) treatment was initiated, and decompressive neurosurgery was performed. The granuloma was removed en bloc and histopathology revealed marked, chronic-active, pyogranulomatous myelitis with intralesional *Coccidioides* spp. spherules with endosporulation. Serum anti-*Coccidioides* spp. antibody titer results revealed a negative IgM and a positive IgG (1:4). The cat was treated with fluconazole for 445 days and examined at various time points, with the last examination 2 years after initial presentation. The cat returned to full ambulation with only mild functional deficits of the right hind limb. In conclusion, this report documents the diagnosis, treatment, and long-term follow up of a cat with a compressive *Coccidioides* spp. spinal cord granuloma. This case highlights the importance of including coccidioidomycosis as a differential diagnosis for cats with peracute hindlimb paraplegia that have lived in or traveled to regions where *Coccidioides* spp. is endemic, and demonstrates the potential for a good long-term outcome with decompressive neurosurgery and antifungal therapy.

## Background

*Coccidioides immitis* and *C. posadasii*, colloquially known as “Valley Fever”, are dimorphic, soil-dwelling fungi that are commonly found in the southwestern United States (1). While most notably endemic to California, Arizona, Texas, Utah, and Mexico, recent studies have discovered *Coccidioides* spp. in the Pacific Northwest, with endemicity identified in Washington state (2, 3). Transmission of *Coccidioides* spp. occurs predominately via inhalation of arthroconidia (infectious spores) that are aerosolized from soil (4). Arthroconidia then enter the pulmonary system where spherulization occurs and can either be eliminated by the host immune response, cause localized respiratory disease, or disseminate via blood and lymphatics to various sites in the body (4).

Coccidioidomycosis is well characterized in dogs, but there is a lack of published information about feline coccidioidomycosis, which is limited to case reports and a few retrospective case series (5–10). The skin is the most common site of dissemination in cats and other less frequently recognized sites include bone, eye, and visceral organs (5, 9, 10). Central nervous system (CNS) infections with *Coccidioides* spp., are not well reported in companion animals, but appear to occur in a minority of cases (1, 5, 8, 10–12). Spinal cord involvement has only been reported in two cats and a clinical description with limited follow-up was provided for just one of those cases (6, 10). Therefore, the objective of the present report was to add to the paucity of available literature with a thorough description of the clinical, advanced imaging, clinicopathologic, surgical, cytologic, and histopathologic features of a cat with spinal cord compression caused by a *Coccidioides* spp., granuloma with long-term follow-up.

## Case Presentation

A 9-year-old neutered male domestic shorthair cat from Maricopa County in Arizona, weighing 4.6 kg (10.1 lbs) was presented to the Companion Animal Clinic at Midwestern University College of Veterinary Medicine (MWU-CVM) for evaluation of a 7-day history of progressive hind limb paraparesis. The cat lived inside the owner's residence and did not venture outside. Seven days before presentation to the MWU-CVM, the cat was evaluated by the primary care veterinarian for a 12-h history of right hind limb paresis. Physical examination revealed decreased patellar and withdrawal (flexor) reflexes of the right hind limb. The remainder of the examination was deemed unremarkable. The cat was administered dexamethasone (0.4 mg/kg subcutaneously once) and discharged with prednisolone (1.6 mg/kg PO q24h) for the purpose of empirically treating a presumed spinal cord disorder with suspected underlying spinal cord edema. The primary care veterinarian evaluated the cat 6 days later, at which time, the left hind limb had become moderately paretic and the right was unchanged. A complete blood count (CBC) and serum chemistry revealed no clinically relevant abnormalities.

**Figure 1:**
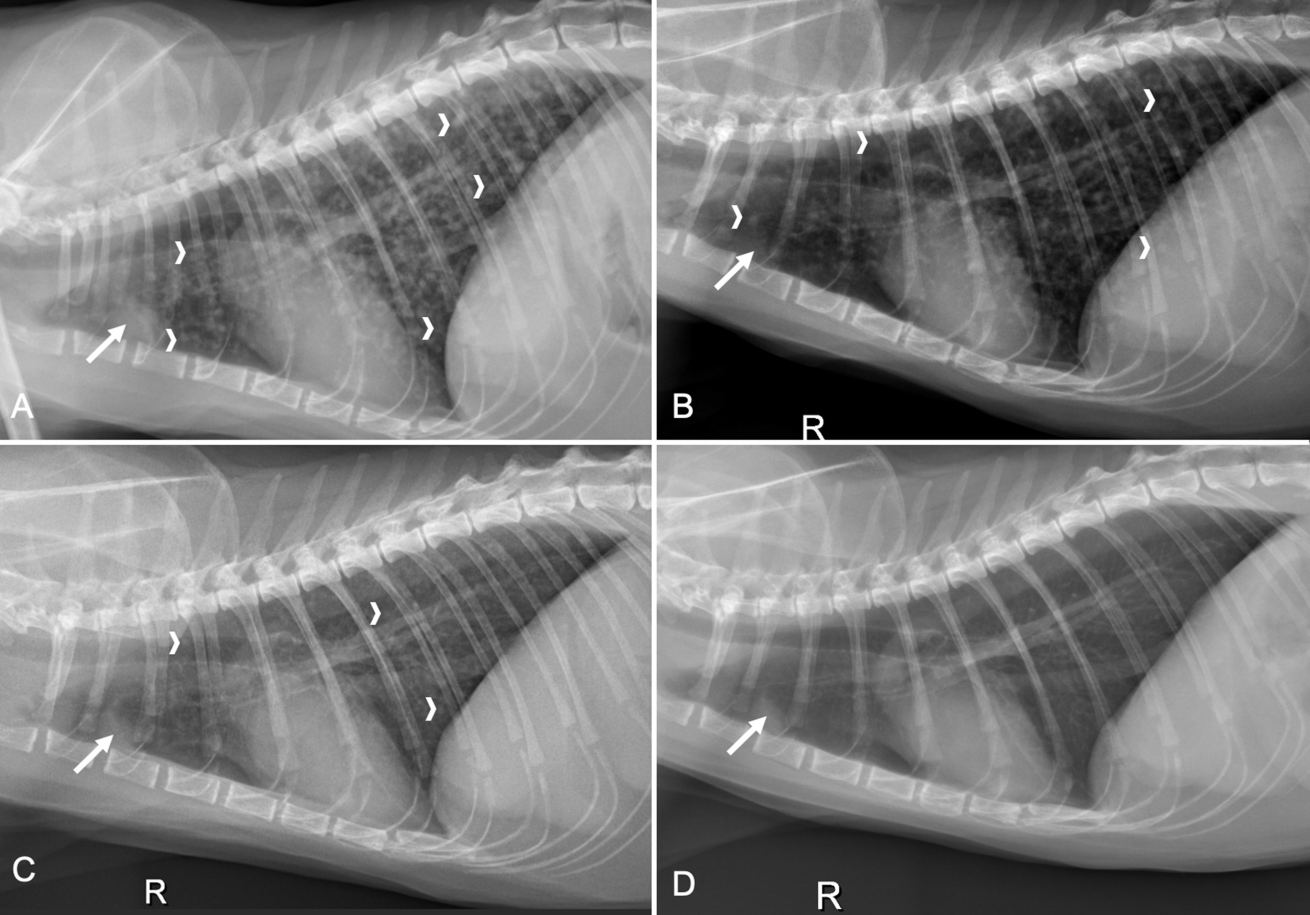
Four right lateral thoracic radiographs obtained from a cat with a compressive *Coccidioides* spp. spinal granuloma from initial presentation and three time points after decompressive neurosurgery and fluconazole therapy. **(A)** Earliest study performed on initial presentation, before surgery, revealed a miliary pulmonary pattern with numerous small soft tissue nodules (white chevrons) in all lung lobes and sternal lymphadenomegaly (solid white arrow); **(B)** On day 35 there were fewer pulmonary nodules (white chevrons) and the sternal lymphadenomegaly remained static (solid white arrow); **(C)** Three months later (day 140), there were even fewer pulmonary nodules (white chevrons) and the sternal lymphadenomegaly remained relatively static (solid white arrow); **(D)** Six months following images in C (day 310), there was resolution of the pulmonary nodules and the sternal lymphadenomegaly was static (solid white arrow).

Physical examination findings upon presentation to the MWU-CVM (day 1), included heart rate of 210 beats/minute, respiratory rate of 30 breaths/minute, and a normal temperature 100.5°F (38.1°C). Cardiothoracic auscultation did not reveal a murmur, arrhythmia, or abnormal lung sounds. There was no history of respiratory abnormalities (e.g., cough, dyspnea, tachypnea, cyanosis). Neurologic examination performed by a board-certified veterinary neurologist (JE) found ambulatory hind limb paraparesis and intact withdrawal reflexes. There was severely decreased voluntary ambulation on the right hind limb and moderate decrease in the left hind limb, with severe gluteal and hamstring muscle atrophy. Hind limb spinal reflexes were intact with increased patellar reflexes. The cutaneous trunci reflex was normal, and the cat vocalized on palpation of L1-L4. The remainder of the examination was unremarkable and neurolocalization supported a T3-L3 myelopathy. Primary differential diagnoses considered included spinal neoplasia (e.g., meningioma or lymphoma), intervertebral disc disease, a vascular event, infectious causes (feline infectious peritonitis, feline leukemia virus (FeLV), coccidioidomycosis, toxoplasmosis), or another inflammatory disorder. Three-view spinal and thoracic radiographs were procured and showed a diffuse miliary pattern (structured interstitial pattern) consisting of numerous small, ill-defined soft tissue nodules (~3 mm) within all lung lobes (Figure 1A). There was also a small broad-based soft tissue structure dorsal to the cranial sternum in the region of the sternal lymph node. The remaining thoracic structures were unremarkable. The lateral radiographs of the lumbar spine showed a widening of the vertebral canal associated with the L4 vertebral body, but there was no permeative/moth-eaten lysis to the bone. A commercial ELISA-based kit (SNAP Feline Triple Test, IDEXX, Westbrook, Minnesota) was negative for detection of antibodies for feline immunodeficiency virus and negative for FeLV and *Dirofilaria immitis* antigen. Tests for *Toxoplasma gondii* and *Coccidioides* spp. IgM and IgG serum antibody titers were submitted (Protatek Reference Laboratory, Mesa, Arizona). The cat was discharged with prednisolone (unchanged dosage) as the only medication.

**Figure 2:**
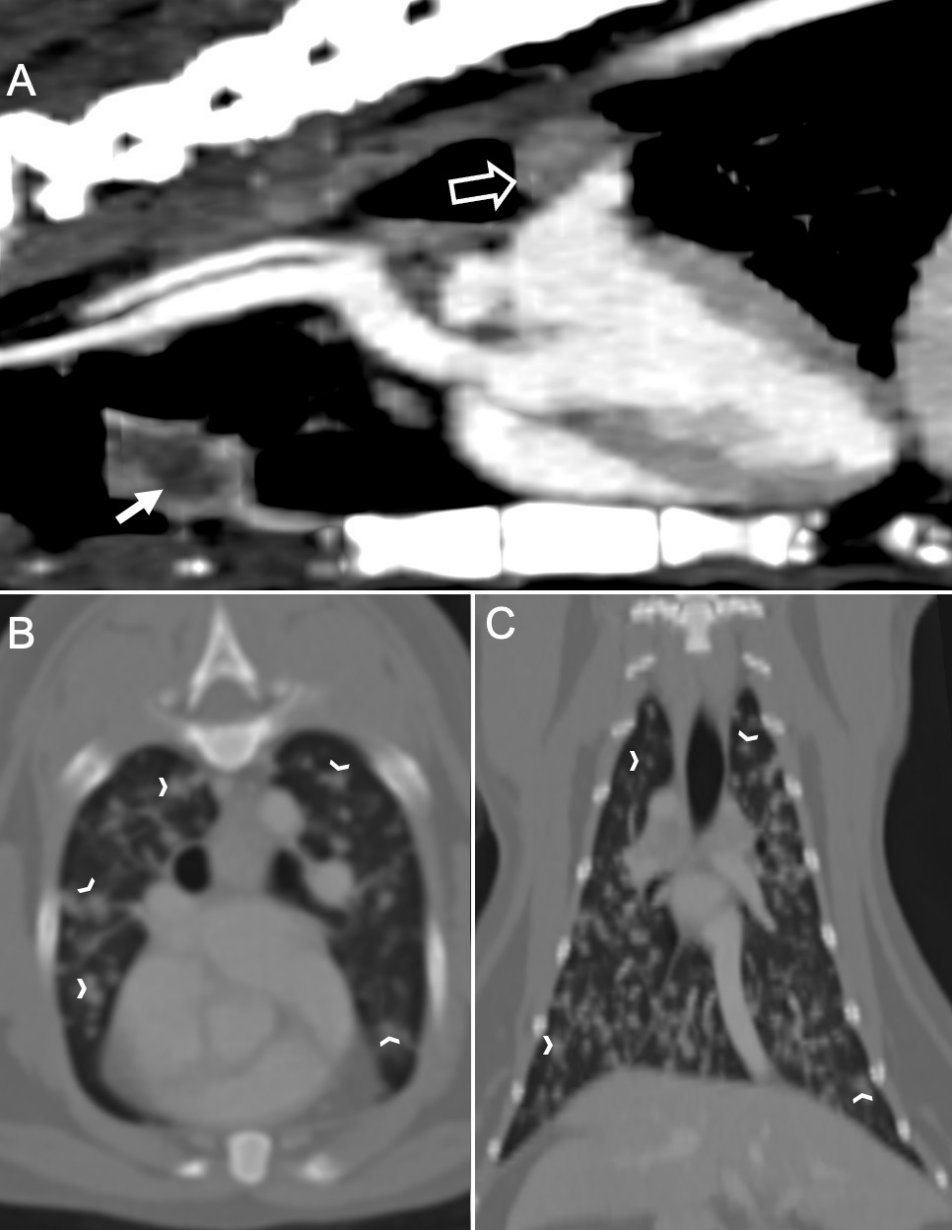
Computed tomography of the thorax in a cat with a compressive *Coccidioides* spp. spinal granuloma before surgery or administration of fluconazole. **(A)** Sagittal plane reconstruction with soft tissue window display. The sternal lymph node was enlarged (sold white arrow) with a peripheral contrast enhancement. There was also enlargement of the tracheobronchial lymph node (hollow white arrow); **(B)** Transverse plane reconstruction with bone window display, and **(C)** Dorsal plane reconstruction with bone window display. There were numerous, irregular, small soft tissue nodules (white chevrons) within all lung lobes.

**Figure 3:**
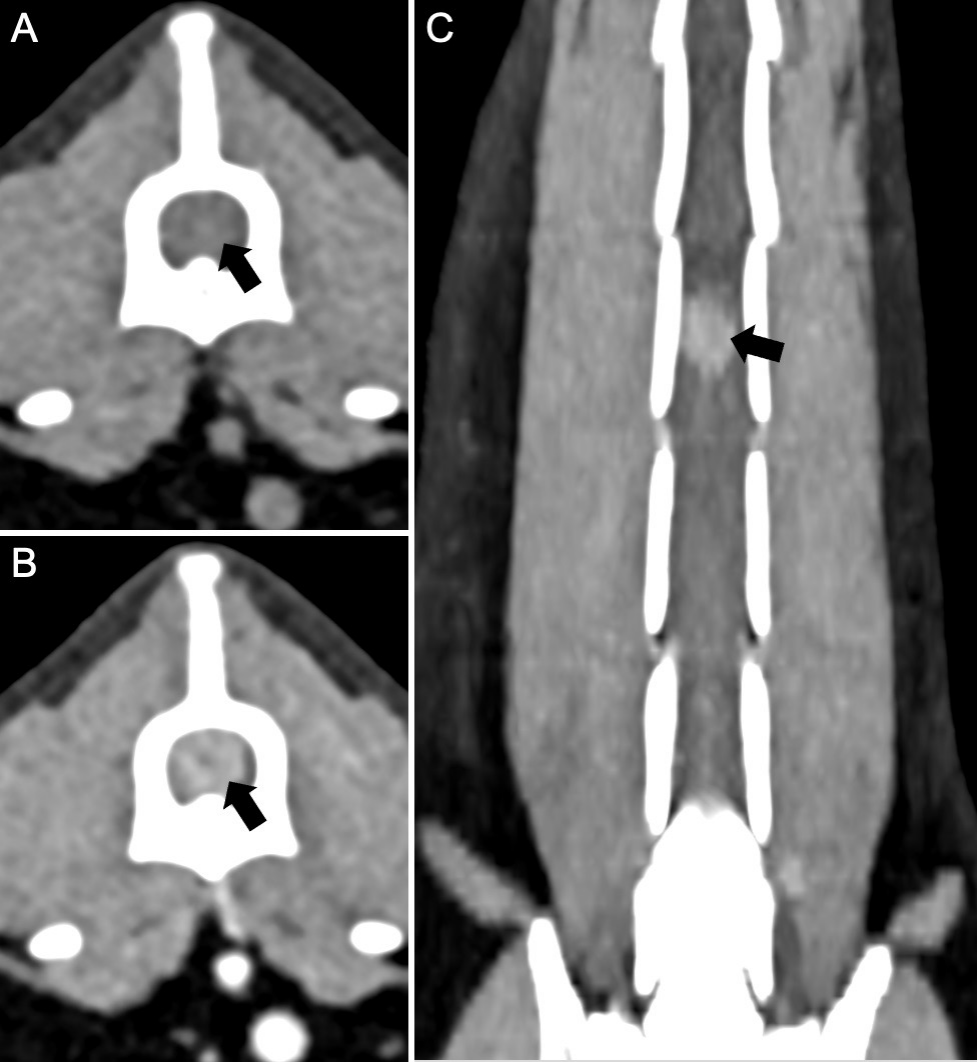
Computed tomography of the lumbar spine in a cat with a compressive *Coccidioides* spp. spinal granuloma before surgery or administration of fluconazole. **(A)** Transverse plane reconstruction, pre-contrast series with soft tissue display. There was enlargement of the spinal cord silhouette (black arrow) that attenuated the epidural fat circumferentially; **(B)** Transverse plane reconstruction, post-contrast series with soft tissue display, and **(C)** Dorsal plane reconstruction, post-contrast series. There was diffuse, strong contrast enhancement of the enlargement of the spinal cord silhouette (black arrow).

The cat was presented again on day 4 for thoracic and spinal computed tomography (CT; CT Scanner, Syngo VC 40 16-slice, Siemens Healthcare, Germany). On examination, voluntary movement of the hind limbs was now absent. The previous paraparesis had progressed to paraplegia with decreased, but present deep pain sensation to both hind limbs. The diffuse miliary pulmonary pattern was confirmed with the CT scan (Figures 2B,C). There was also mild enlargement of the tracheobronchial lymph nodes, cranial mediastinal lymph nodes, and sternal lymph node (Figure 2A). In the pre-contrast series, there was an isoattenuating widening of the spinal cord silhouette dorsal to the vertebral body of L4 (Figure 3A). The focal widening had a diffuse, homogeneous enhancement with distinct borders. There was an expansile widening of the spinal canal dorsal to L4 associated with the strongly contrast-enhancing mass (Figures 3B,C).

**Figure 4:**
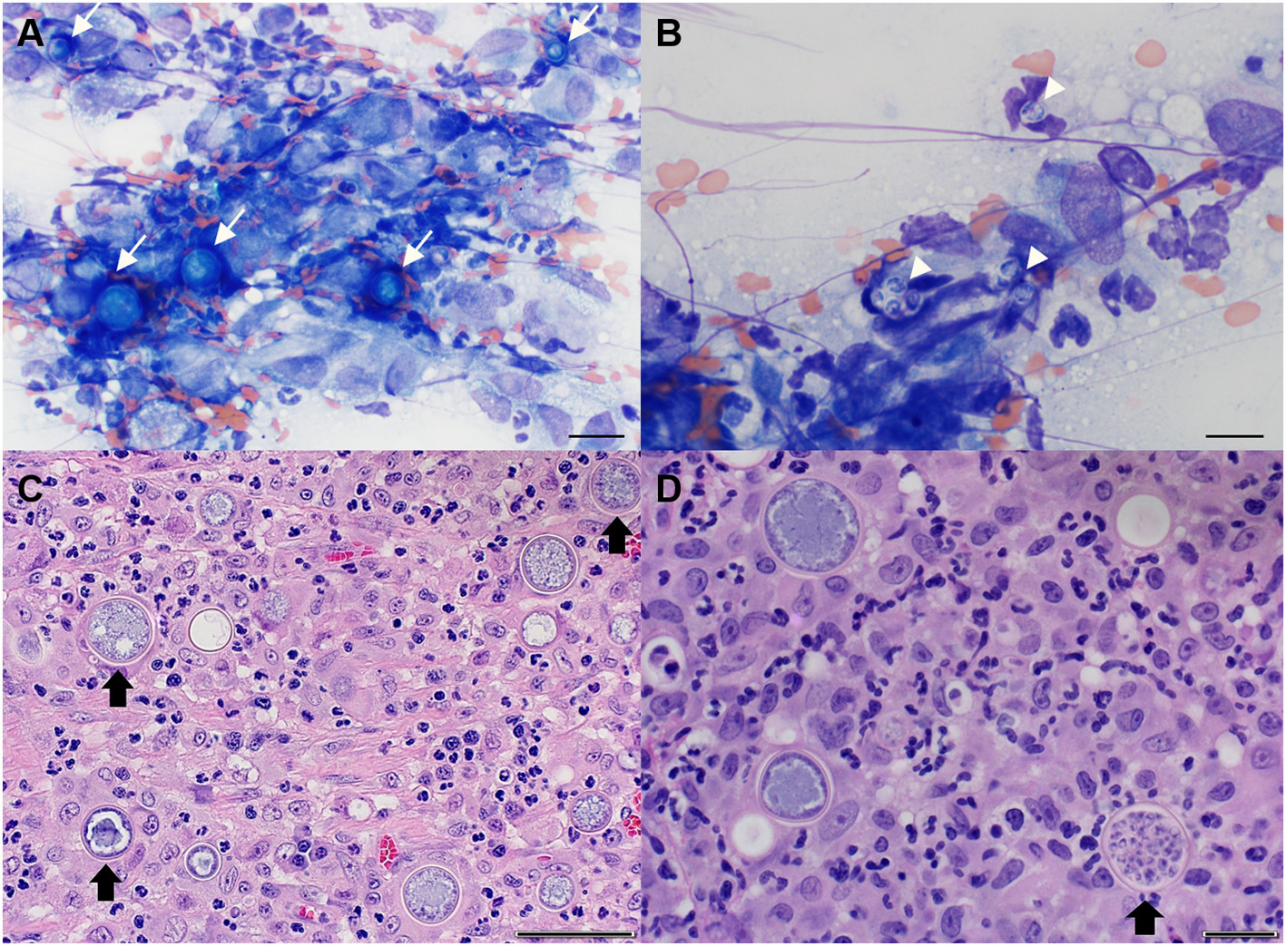
Representative cytological images of sternal lymph node **(A,B)** and histopathological images of spinal cord granuloma **(C,D)** in a cat with spinal coccidioidomycosis. **(A)** Multiple immature *Coccidioides* spp. spherules (arrows) with surrounding pyogranulomatous inflammation. Wright-Giemsa, 50× objective, Scale bar = 20 μm; **(B)** Several free *Coccidioides* spp. endospores (arrowheads). Wright-Giemsa, 100× objective, Scale bar = 10 μm; **(C)** Neuroparenchyma obscured by mixed inflammation predominated by neutrophils and macrophages admixed with numerous *Coccidioides* spp. spherules (arrow). Hematoxylin and eosin (H and E). Scale bar = 50 μm; **(D)** Occasional *Coccidioides* spp. spherules are filled with endospores (arrow) (H and E). Scale bar = 20 μm.

Cytologic interpretation of samples obtained via fine needle aspiration of the lung and sternal lymph node revealed pyogranulomatous inflammation with *Coccidioides* spp. spherules and endospores (Figures 4A,B). Cisternal cerebral spinal fluid (CSF) was examined cytologically and revealed a nucleated cell count of 1 cell/μL (normal, <8 cells/μL), a red blood cell count of 2,470 cells/μL (reference, 0 cells/μL), and a total protein of 22.5 mg/dL (reference, <25 mg/dL). A total of 12 nucleated cells were present on the cytospin preparation and consisted solely of non-degenerative neutrophils. There were no infectious agents or evidence of neoplasia observed. The small amount of red blood cells in the CSF sample likely represented a slight blood contamination from the collection process and was not considered significant. Based upon the cytologic results from the lung lobe and sternal node, a suspected diagnosis of a spinal *Coccidioides* spp. granuloma was made. Magnetic resonance imaging (MRI) of the thoracolumbar spinal cord with emergent surgical decompression at a veterinary referral hospital with MRI capabilities was recommended, but the owner declined and elected to have an exploratory spinal cord decompressive procedure performed at the MWU-CVM. The cat was discharged with the following medications: fluconazole (10.9 mg/kg PO q12h) and prednisolone (unchanged dosage).

Scheduling conflicts resulted in the cat being presented for surgery 3 days later (day 7). Immediate surgical decompression is generally recommended for paraplegic patients or those with acute rapid deterioration of myelopathic signs as delay in surgical intervention could result in a poor prognosis (13). The neurological examination at presentation for surgery was unchanged from the previous evaluation. A right hemilaminectomy/spinal exploratory surgery at mid-body of L4 was performed (Supplemental Figure 1). No extradural lesion was present upon opening the window, but the spinal cord was focally swollen and discolored over the midbody of L4 (Supplemental Figure 1A). A durectomy was performed over the exposed spinal cord and revealed a swollen and discolored spinal cord segment (Supplemental Figure 1B). A myelotomy was performed that revealed an intra-medullary mass (Supplemental Figure 1C). The mass was approximately 0.75 cm, round, semi-firm, and was removed en bloc (Supplemental Figure 1D). The spinal cord ventral to the mass was bruised and substantially compressed, leaving an indent that gradually re-expanded as the mass was removed (Supplemental Figure 1D). The overall dark purple coloring of the spinal cord gradually improved (Supplemental Figure 1E). A small amount of dark coloration and hemorrhage remained at the center of the residual indentation. It was undetermined if the remaining indentation was due to compression from the mass or a focal area of myelomalacia. The excised mass was submitted for histopathology. The cat was hospitalized overnight with an indwelling urinary catheter. Treatments included: fentanyl (3 μg/kg IV CRI), Lactated Ringers solution (12 mL/h), dexamethasone (0.22 mg/kg IV q24h), cefazolin (22 mg/kg IV q8h), fluconazole (10.9 mg/kg PO q12h), and 7.2% NaCl (5 mL/kg IV q6h). Hypertonic saline was administered because it accelerates the reduction of spinal cord edema and stabilizes neuronal function in cases of acute, severe spinal cord injury (14, 15).

The day after surgery (day 8), the urinary catheter was removed. Minimal voluntary movement was noted in the right hind limb with good voluntary movement in the contralateral limb. Postural reactions were decreased in both hind limbs, but were improved compared to before surgery when they were absent. The following day (day 9), the cat was able to use the left hind limb to stand and ambulate, but there was still minimal movement in the right hind limb though, there was improvement in muscle tone. There was normal tactile placing and proprioception in the left hind limb with absent postural reactions in the right. Histopathology of the excised mass showed that normal spinal cord architecture was obscured by mixed inflammation dominated by epithelioid macrophages, few multinucleated giant cells, neutrophils, and fewer, often perivascular, lymphocytes and plasma cells. Admixed within the inflammatory cells were frequent hyperemic reactive vessels and numerous circular spherules that measured up to 33 μm in diameter with a thin 1 μm refractile capsule that surrounded combinations of central basophilic stippling, vacuolation, and packed with 2–3 μm diameter endospores (consistent with *Coccidioides* spp.). Occasionally, the spherules were degenerate with externally released endospores. At the periphery of sections, residual neuroparenchyma contained gemistocytic astrocytes, acute hemorrhage, degeneration, and malacia (Figures 4C,D). Serologic test results for *Toxoplasma gondii* (IgM and IgG negative) and *Coccidioides* spp. (IgM negative, IgG 1:4) also returned. The cat was discharged (2 days after surgery) on day 10, at which time, there had been considerable improvement. The cat was mobile using the left hind limb, which demonstrated moderate paresis. The right hind limb had good extensor tone, but still was without voluntary movement. The medications prescribed at discharge included fluconazole (unchanged dosage) and prednisolone (gradually weaned and discontinued 14 days later).

The cat was presented for planned evaluations on days 20 and 35. The surgical incision was unremarkable on day 20. Neurologic findings at both examinations were similar; the cat was ambulatory in the hind limbs with a mild to moderate hind limb ataxia and right hind limb paresis. Postural reactions to the left hind limb were nearly normal but delayed to the right hind limb. Spinal reflexes to the left hind limb were normal, but the withdrawal reflex to the right hind limb was mildly decreased. The cutaneous trunci reflex was normal and the cat was only mildly reactive on spinal palpation over the surgery site (Supplemental Video 1).

A serum chemistry, CBC, *Coccidioides* spp. antibody titers (Protatek Reference Laboratory) and thoracic radiographs were performed on day 35. The *Coccidioides* spp. IgM remained negative and there was a one-fold reduction in IgG titer (1:2). Thoracic radiographs revealed an improvement to the miliary pulmonary pattern with static sternal lymphadenomegaly (Figure 1B). The CBC and serum chemistry were unremarkable. Medications at the time of discharge included fluconazole (unchanged dosage) and gabapentin (3.0 mg/kg PO q12h, as needed for pain).

On day 140, the cat was presented for a planned evaluation and the owner reported the cat had become fully ambulatory and recently started to climb a “cat tree” in the home. Neurologic examination revealed mild ataxia in the right hind limb with slight toe-dragging. The postural reactions in the right hind limb were still decreased (Supplemental Video 2). Serum chemistry, CBC, *Coccidioides* spp. antibody titers, urinalysis, and thoracic radiographs were performed. The *Coccidioides* spp. titers remained similar (IgM negative, IgG 1:4) (Antech Diagnostics, Irvine, California) and the CBC, chemistry, and urinalysis were unremarkable. Thoracic radiographs revealed improvement of the miliary pattern with fewer pulmonary nodules and the remaining soft tissue nodules were less distinct. The sternal lymphadenomegaly was still identifiable and similar to the previous study (Figure 1C). Fluconazole was continued unchanged and gabapentin was discontinued 30 days later.

The cat was presented for an additional recheck evaluation on day 310. The owner reported that since the last examination, the cat ambulated normally, was able to jump to the second tier of the “cat tree”, and run, but occasionally limped on the right hind limb. Neurologic examination revealed continued mild improvement with ambulation to both hind limbs with mild paresis of the right hind limb that occasionally “knuckled” while walking. Postural reactions on the right hind limb were slightly delayed but normal in the remaining three limbs (Supplemental Video 3). Serum chemistry, CBC, *Coccidioides* spp. antibody titers (Antech Diagnostics), urinalysis, and thoracic radiographs were performed. The *Coccidioides* spp. titers were negative and the CBC and urinalysis were unremarkable. There was a mild increase in serum alanine transaminase activity (ALT; 196 IU/L, reference interval chemistry, 10–100 IU/L); all other measured chemistry analytes were unremarkable. Fluconazole was decreased (12 mg/kg PO q24h) and then discontinued approximately 4 months later (day 452). Thoracic radiographs at this time showed resolution of the pulmonary nodules (Figure 1D). A recheck assessment performed on day 463 revealed persistent proprioceptive ataxia and decreased postural reactions of the right hind limb (Supplemental Video 4). *Coccidioides* spp. antibody titers (Protatek Reference Laboratory) were similar (IgM negative; IgG 1:2). No medications were prescribed at discharge.

The last in-hospital recheck examination took place on day 739 (2 years). The owner reported that although there was some decrease in function of the right hind limb, it did not hinder normal activity. There were no systemic signs of illness that could be associated with *Coccidioides* spp infection (e.g., altered appetite and lethargy). On examination, the cat was ambulatory with a moderate shuffling gait associated with the right hind limb, with occasional knuckling. Postural reactions and spinal reflexes to the right hind limb were delayed to absent. Evaluation of the left hind limb was unremarkable (Supplemental Video 5). The functional deterioration of the right hind limb was within the spectrum of reasonable expectation 2 years after a severe spinal cord injury. *Coccidioides* spp. antibody titers (Protatek Reference Laboratory) were repeated and were unchanged from previous results (IgM negative; IgG 1:2). Given there were no significant clinical changes, other than what might be expected over time, no specific treatment was recommended.

## Discussion

*Coccidioides* spp. infections begin with inhalation of aerosolized arthroconidia that grow from mycelia in the soil (4). Arthroconidia disperse along the bronchial tree into the alveoli and develop into immature spherules that mature, grow in size, and undergo endosporulation (4). Eventually, first generation spherules rupture and release endospores that elicit a profound host inflammatory response characterized by an influx of polymorphonuclear cells and macrophages (4). These endospores mature into second generation spherules that serve to perpetuate this cycle in the host. Approximately 60% of humans that inhale *Coccidioides* spp. arthroconidia develop infections that manifest with mild to no reportable symptoms (16, 17). This was evident in our case as there were no reported respiratory signs despite obvious abnormalities identified in thoracic imaging studies. A recent study suggest the potential for similar rates of subclinical infections in dogs from Arizona; however, similar serosurvey studies have not been performed in cats (18). Dissemination occurs when endospores spread via lymphatics and blood to extra-pulmonary sites. This complication of infection occurs in 1% and 25% of humans and dogs, respectively (1, 17). The frequency of occurrence has not been investigated in cats; although, anecdotal review of available published case series suggest it could be similar to dogs (5, 10). The most commonly reported dissemination site in cats is the dermis and subcutis (5, 9, 10). Central nervous system coccidioidomycosis is uncommonly reported in cats and only two cases of granuloma-associated spinal cord compression in addition to the case reported here have been published (6, 10).

The cat in this report was evaluated for progressive hind limb paraplegia with initial neurolocalization supportive of a T3-L3 myelopathy despite the site of the granuloma being found over the L4 vertebral body. The T3-L3 localization refers to the suspected spinal cord segment involved based on clinical findings and does not directly correlate with the corresponding vertebral body (19). Several relevant infectious diseases were excluded with serologic diagnostic test results. Coccidioidomycosis was considered a possible differential diagnosis because the cat lived in Arizona. Several diagnostic tests were performed to interrogate this and other differential diagnoses, including anti-*Coccidioides* spp. antibody serology, thoracic and spinal CT, transthoracic lung and sternal lymph node aspiration with cytologic review, CSF cytologic evaluation, and histopathologic assessment of the compressive granuloma. Collectively, this assortment of diagnostic tests highlights the difficulty in establishing a definitive diagnosis of coccidioidomycosis in some cases (20).

Diagnosis of *Coccidioides* spp. infection usually includes serological testing for antibodies using agar gel immunodiffusion (AGID) (20). Coccidioidal serology is useful in determining that a cat has been infected, but has its limitations, as were apparent in the current report. Serologic positivity reflects current or historical infection, but does not necessarily confirm *Coccidioides* spp. is the cause for the patient's illness, especially with low IgG titers ( ≤ 1:8). Shubitz et al. (18) revealed in a seroprevalence study that healthy dogs in endemic regions can have quantitative IgG titers up to 1:16. Further obscuring the association between serology and disease is that a negative titer result does not rule out infection (20). The difficulty in interpreting the clinical relevance of anticoccidioidal antibody titers was evident in the case reported here. The baseline anticoccidioidal IgM was negative, and the IgG titer was low positive (1:4). Therefore, we could not decipher if the low anticoccidioidal antibody positivity was related to a current or historical infection. Another obstacle associated with coccidioidal serologic testing is that commercial laboratories often take up to 7–10 days to report AGID results. This was evident in our case, as antibody titer results returned 9 days after the original submission. The identification of *Coccidioides* spp. spherules by histopathologic and/or cytologic examination is diagnostic, specific, and is generally reported faster than serologic testing, but have variable sensitivities due to the typically limited abundance of intralesional *Coccidioides* spp. organisms (11, 21). Cytologic examination of fine needle aspirations from lung lobe and sternal lymph node provided a rapid confirmation that this cat had evidence of intrathoracic coccidioidomycosis; however, this information alone was insufficient to establish a definitive connection to the compressive spinal lesion identified on CT. Ultimately, histopathologic examination of the spinal granuloma was required to make a definitive diagnosis. While cytology results could not provide a definitive diagnosis of the spinal cord lesion, it did allow for rapid initiation of antifungal therapy for the confirmed intrathoracic coccidioidal infection.

The characteristics of the spinal granuloma on CT were similar to MRI findings in cases of solitary CNS granulomas, as reported in Bentley et al. (8), where differentiation between intra-axial and axial masses was difficult. Granulomas share similar features with neoplastic masses, and extra-axial granulomas often lack a distinct border between the mass and neural parenchyma. In this case, it was impossible to determine if the mass was intra-medullary or not, suggesting that CT is helpful in determining the granuloma location, but not in differentiating exact spinal involvement. The lack of eccentric deviation of the spinal cord within the spinal canal in multi-planar reformats and the concentric dilation of the spinal cord silhouette with circumferential attenuation of the epidural fat supports an intramedullary differentiation from both extramedullary-intradural and extradural localizations. Intramedullary contrast enhancement of a mass is not a specific sign for infectious granulomas, as they share imaging characteristics with neoplasms such as lymphoma (22). The overlap in advanced imaging features between spinal *Coccidioides* spp. granulomas and neoplastic masses is important because it reinforces that coccidioidomycosis should remain a differential diagnosis in cats that have lived in or traveled to regions where *Coccidioides* spp. is endemic.

Treatment information for spinal *Coccidioides* spp. granulomas in companion animals are limited in the literature (6). In our case, decompressive surgery was met with a good clinical outcome. The cat returned to full ambulation of the hind limbs, with only mild deficits noted in the right hind limb 2 years after surgery. The total duration of fluconazole treatment lasted 445 days, and the miliary pulmonary pattern initially identified on thoracic radiographs resolved 310 days after initial presentation. The decision to discontinue fluconazole administration after 445 days was made after a collaborative assessment of a prolonged subclinical status, negative to weakly positive anticoccidioidal IgG titers on serial evaluations, resolution of thoracic radiographic abnormalities, and the cat had received antifungal treatment for more than a year. Specific evidence-based treatment recommendations for disseminated coccidioidomycosis in companion animals are lacking. The authors', and others, generally treat animals with disseminated coccidioidomycosis for a minimum of 1 year in conjunction with resolution of clinical signs and imaging abnormalities, plus reduction in serum anticoccidioidal IgG titer to ≤ 1:4 (1). Similar to the results presented here, another cat with a compressive *Coccidioides* spp. spinal granuloma treated with surgery and fluconazole reported in Foureman et al. (6) greatly improved at the last follow-up evaluation 6 weeks after surgery.

## Conclusion

In conclusion, this report documents the diagnosis, treatment, and long-term follow up of a cat with a compressive *Coccidioides* spp. spinal cord granuloma. This case highlights the importance of including coccidioidomycosis as a differential diagnosis for cats with peracute hindlimb paraplegia that have lived in or traveled to regions where *Coccidioides* spp. is endemic. This report also serves as an indication that cats with compressive *Coccidioides* spp. spinal granulomas have potential for a good long-term prognosis with decompressive surgery and fluconazole therapy.

## Data Availability Statement

The original contributions presented in the study are included in the article/Supplementary Material, further inquiries can be directed to the corresponding author/s.

## Ethics Statement

Ethical review and approval was not required for the animal study because this case study used historical medical records and did not require owner consent.

## Author Contributions

HD writing and editing of the manuscript and review of final submission. JE management of case, collection of data, writing and editing of the manuscript, figure preparation, and review of final submission. JJ, KW, JS, and EH writing and editing of the manuscript, figure preparation, and review of final submission. All authors contributed to the article and approved the submitted version.

## Conflict of Interest

The authors declare that the research was conducted in the absence of any commercial or financial relationships that could be construed as a potential conflict of interest.

## Publisher's Note

All claims expressed in this article are solely those of the authors and do not necessarily represent those of their affiliated organizations, or those of the publisher, the editors and the reviewers. Any product that may be evaluated in this article, or claim that may be made by its manufacturer, is not guaranteed or endorsed by the publisher.

## Supplementary Material

Supplementary Video 5
www.kaggle.com/dataset/05b4d3bfd04569f526ea831f6a9df78c616819b648852a32e925315a6ae68c8f

Supplementary Figure 1 Representative intraoperative images from a cat with a compressive *Coccidioides* spp. spinal granuloma. **(A)** Post hemilaminectomy and durectomy. There was no extradural or intradural-extramedullary pathology but the spinal cord was edematous and discolored (open arrow); **(B)** Immediately post myelotomy. The spinal cord was edematous with suggestion of intramedullary disease (open arrow); **(C)** Post myelotomy. With initial exploration, the granuloma was partially extruding from within the cord parenchyma (open arrow); **(D)** Post myelotomy. The granuloma was fully removed from within the cord parenchyma (open arrow), but the cord appeared discolored with the possibility of focal necrosis (closed arrow); **(E)** the discoloration of the spinal cord dissipated after several minutes (closed arrow); **(F)** Close-up of the removed granuloma measuring approximately 0.75 cm in diameter.
