# Supplementary figures and images for: Case Report: Successful Management of a Compressive Intraspinal Coccidioides Species Granuloma in a Cat
Source: Front Vet Sci. 2022 Jan 3;8:801885. doi: 10.3389/fvets.2021.801885 (PMC8757464; doi:10.3389/fvets.2021.801885)

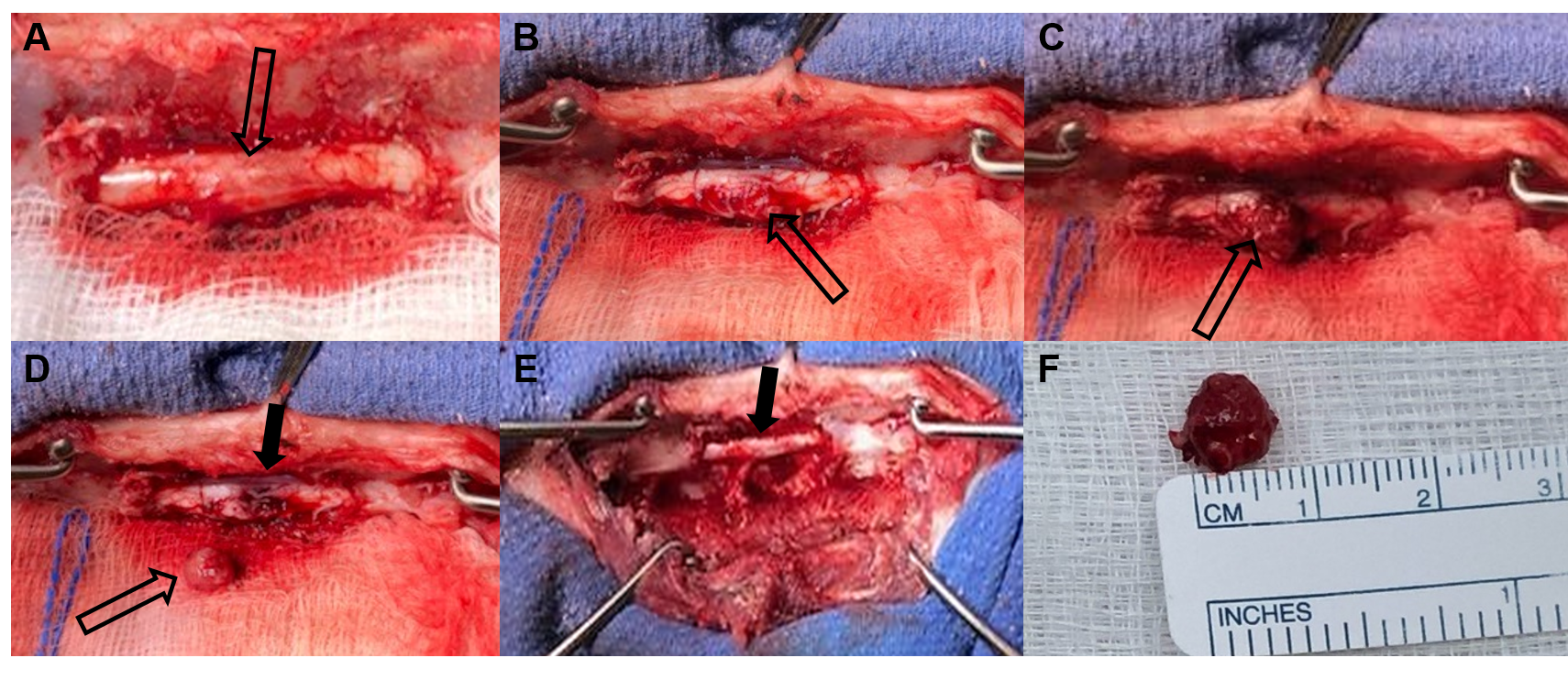

Supplement: Supplementary Figure 1 — Representative intraoperative images from a cat with a compressive Coccidioides spp. spinal granuloma. (A) Post hemilaminectomy and durectomy. There was no extradural or intradural-extramedullary pathology but the spinal cord was edematous and discolored (open arrow); (B) Immediately post myelotomy. The spinal cord was edematous with suggestion of intramedullary disease (open arrow); (C) Post myelotomy. With initial exploration, the granuloma was partially extruding from within the cord parenchyma (open arrow); (D) Post myelotomy. The granuloma was fully removed from within the cord parenchyma (open arrow), but the cord appeared discolored with the possibility of focal necrosis (closed arrow); (E) the discoloration of the spinal cord dissipated after several minutes (closed arrow); (F) Close-up of the removed granuloma measuring approximately 0.75 cm in diameter. [file Image_1.TIF]
